# The human allicin-proteome: *S*-thioallylation of proteins by the garlic defence substance allicin and its biological effects

**DOI:** 10.1016/j.freeradbiomed.2018.11.022

**Published:** 2019-02-01

**Authors:** Martin C.H. Gruhlke, Haike Antelmann, Jörg Bernhardt, Veronika Kloubert, Lothar Rink, Alan J. Slusarenko

**Affiliations:** a Department of Plant Physiology, RWTH Aachen University, Worringer Weg 1, D-52056 Aachen, Germany; b Freie Universität Berlin, Institute of Biology-Microbiology, Königin-Luise-Str. 12-16, D-14195 Berlin, Germany; c Institute of Microbiology, University of Greifswald, Felix-Hausdorff-Straße 8, D-17489 Greifswald, Germany; d Institute of Immunology, RWTH Aachen University Hospital, Pauwelsstraße 30, D-52074 Aachen, Germany

**Keywords:** Allicin, *S*-thioallylation, Protein modification, Cysteine, Cytoskeleton, Actin, Glycolysis, Warburg effect, Enolase, Zinc, Jurkat, Fibroblasts, T-cells, Interleukin IL-1, IL-2

## Abstract

A single clove of edible garlic (*Allium sativum* L.) of about 10 g produces up to 5 mg of allicin (diallylthiosulfinate), a thiol-reactive sulfur-containing defence substance that gives injured garlic tissue its characteristic smell. Allicin induces apoptosis or necrosis in a dose-dependent manner but biocompatible doses influence cellular metabolism and signalling cascades. Oxidation of protein thiols and depletion of the glutathione pool are thought to be responsible for allicin's physiological effects. Here, we studied the effect of allicin on post-translational thiol-modification in human Jurkat T-cells using shotgun LC-MS/MS analyses. We identified 332 proteins that were modified by *S*-thioallylation in the Jurkat cell proteome which causes a mass shift of 72 Da on cysteines. Many *S*-thioallylated proteins are highly abundant proteins, including cytoskeletal proteins tubulin, actin, cofilin, filamin and plastin-2, the heat shock chaperones HSP90 and HSPA4, the glycolytic enzymes GAPDH, ALDOA, PKM as well the protein translation factor EEF2. Allicin disrupted the actin cytoskeleton in murine L929 fibroblasts. Allicin stimulated the immune response by causing Zn^2+^ release from proteins and increasing the Zn^2+^-dependent IL-1-triggered production of IL-2 in murine EL-4 T-cells. Furthermore, allicin caused inhibition of enolase activity, an enzyme considered a cancer therapy target. In conclusion, our study revealed the widespread extent of *S*-thioallylation in the human Jurkat cell proteome and showed effects of allicin exposure on essential cellular functions of selected targets, many of which are targets for cancer therapy.

## Highlights

• Allicin from garlic caused *S*-thioallylation of 332 proteins in the human Jurkat cell proteome.
• Main *S*-thioallylated proteins are cytoskeletal proteins, chaperones, glycolytic enzymes, translation factors.
• Selected *S*-thioallylated proteins were shown to be inhibited by allicin.
• Allicin disrupted the cytoskeleton, decreased enolase activity and enhanced Zn^2+^ release.
• Mode of action of allicin in mammalian cells could explain its cytostatic effect in cancer cells.

## Graphical abstract

**Figure:**
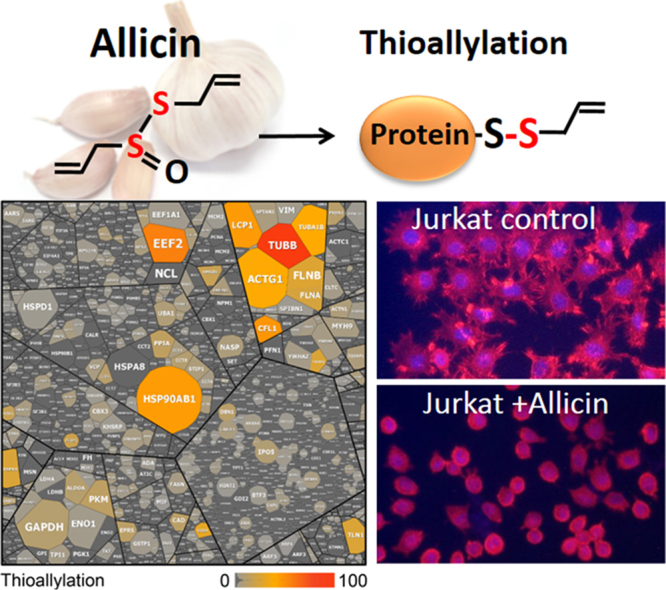

## 1 Introduction

**Fig. 1:**
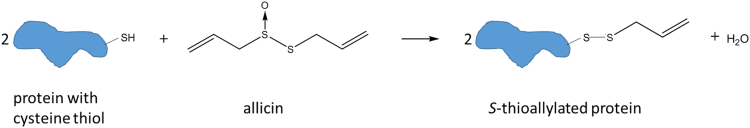
*S*-thioallylation of cysteine thiols in proteins by allicin. This leads to a mass increase of 72 Da at Cys residues in proteins.

Alliums, such as onions, garlic, shallots, chives *etc*. are an integral part of culinary repertoires world-wide and a diet without them would be less flavourful. Alliums are well known for the characteristic pungent aromas which develop when the plants are damaged. In addition to being tasty, many *Allium* spp., including garlic and its extracts, have been used in medicines since ancient times. For example, an Egyptian medical papyrus, the Codex Ebers from around the 16th century B.C., lists 22 preparations which contain garlic. The antiseptic and antibiotic properties of garlic are well documented, and garlic has been used in folk-medicine for treating wounds and infections in people and animals [1]. Upon damage of garlic cells, the enzyme alliinase (E.C. 4.4.1.4) is released from the vacuole to the cytoplasm producing allicin (diallylthiosulfinate) from alliin (*S*-allyl-L-cysteine sulfoxide). Allicin is the first and main sulfur compound and a single clove of garlic of about 10 g from a composite bulb releases up to 5 mg of allicin [2]. Allicin was chemically characterized and identified as the major antibacterial substance from garlic by Cavallito and Bailey in 1944 [3], [4]. Allicin decomposes readily to 2-propenesulfenic acid and 2-propenethial (thioacrolein), which enter into a cascade of reactions producing alkyl disulfides, including 3-[(Prop-2-en-1-yl)disulfanyl]prop-1-ene (diallyl disulfide) and various polysulfanes, vinyl dithiins, and ajoene. Many of these substances are physiologically active [5]. Allicin exhibits a broad range of antimicrobial activities against multi-drug-resistant bacteria, fungi and oomycetes [6], [7], [8]. Allicin readily permeates cell membranes and is dose-dependently toxic to mammalian cells. Allicin induces apoptosis and inhibits cell proliferation at sublethal doses [9], [10], [11], [12], [13], [14]. Allicin was reported early on to inhibit tumour growth in rats [15] and the anticancer effects of allicin have been confirmed repeatedly [16], [17], [18], [19]. Indeed, a strategy to generate allicin *in situ* within tumour tissue was shown to be effective against a human tumour cell line xenograft in athymic nude mice, while at the same time leaving other tissues unharmed [20]. At biocompatible concentrations allicin affects signalling pathways and gene expression, resulting in alteration of the physiological status of cells [21], [22], [23]. Since allicin reacts rapidly with thiol-groups in cysteine, it has been proposed that the inhibitory and toxic effects are due to inactivation of crucial enzymes of central metabolism [3], [24]. Allicin reacts with Cys thiol residues to form mixed disulfides by *S*-thioallylation (Fig. 1). In *E. coli*, *S*-thioallylation was shown to be the predominant form of allicin-induced protein oxidation in the proteome [25]. The *S*-thioallylation of some specific eukaryotic proteins upon allicin treatment has been shown for haemoglobin [26] and peroxiredoxin [27].

In this study we characterize the allicin-proteome of human Jurkat cells, which are an immortalized line of human T-lymphocyte cells that are used to study T-cell signalling and the susceptibility of cancers to drugs [28]. Jurkat cells were treated with a biocompatible dose of allicin and we demonstrated biological effects on selected targets identified as *S*-thioallylated using mass spectrometry-based proteomics analysis.

## 2 Materials and methods

### 2.1 Cell culture

The Jurkat T-cell line and L929 fibroblasts were cultured in RPMI 1640 (Sigma-Aldrich, Steinheim, Germany) medium supplemented with 10% heat-inactivated FCS (PAA; Germany), 2 mM L-glutamine, 100 U/mL of penicillin, and 100 µg/mL of streptomycin, additionally supplemented with 1% 100x non-essential amino acids and 1% 100 mM sodium pyruvate (all Sigma-Aldrich). Cells were maintained at 37 °C, 100% humidity, and 5% CO_2_.

EL-4 cells were also cultivated in RPMI 1640 medium, supplemented with 5% FCS, 2 mM L-glutamine, 100 U/mL penicillin and 100 µg/mL streptomycin.

### 2.2 MTT-assay for cell cellular metabolic activity

Allicin was diluted in RPMI 1640 medium to the concentrations as indicated. 50 µL of allicin was mixed with 50 µL cell suspension (in RPMI 1640) in wells of 96-well plates (Sarstedt, Nuembrecht, Germany). All treatments were performed in triplicate. The final cell number was 1 × 10^5^ cells per well. Cells were incubated for three days at 37 °C and 5% CO_2_. 50 µL MTT (3-(4,5-dimethylthiazol-2-yl)-2,5-diphenyltetrazolium bromide, Carl Roth GmbH, Karlsruhe, Germany, 1% w/v in PBS) was added and incubated for three hours at 37 °C and 5% CO_2_. Cells were collected by centrifugation (300×*g*), washed with PBS and the supernatant was removed. 100 µL 2-propanol was added and plates were incubated for 15 min under shaking to solve the formazan salt. The measurement was performed at 570 nm in a microplate reader (Sunrise plate reader, Tecan, Crailsheim, Germany.)

### 2.3 Synthesis of allicin

Allicin was synthezised by oxidizing 3-[(Prop-2-en-1-yl)disulfanyl]prop-1-ene (diallyl disulfide) with peracetic-acid (glacial acetic acid/H_2_O_2_) as previously described [11]. 3-[(Prop-2-en-1-yl)disulfanyl]prop-1-ene (diallyl disulfide - DADS, Sigma-Aldrich, Steinheim, Germany) was distilled under vacuum before use and purity was checked with HPLC. DADS (2 g = 14 mmol) was dissolved in 5 mL glacial acid (Carl Roth, Karlsruhe, Germany) and 3 mL of ice-cold 30% hydrogen peroxide (Merck, Darmstadt, Germany) was added dropwise. The reaction was continued for 30 min on ice and subsequently the temperature of the reaction mixture was allowed to increase to room temperature and stirred for an additional two hours. The reaction was stopped by adding 25 mL deionized water (18.2 MΩ cm−1) and was extracted twice with each 30 mL dichloromethane (Carl Roth, Karlsruhe, Germany). Acetic acid was removed by washing the extract several times with an aqueous 5% (wt/v) NaHCO_3_ solution. Subsequently, the extract was washed with distilled water until pH 6–7 was reached. The organic phase was evaporated in vacuo and the oil obtained was dissolved in 200 mL distilled water. The purity of the reaction product was checked by HPLC. If unreacted DADS was detected, an extraction with 0.1 vol% hexane was used to remove residues of DADS. Allicin was subsequently extracted with CH_2_Cl_2_, dried over anhydrous MgSO_4_ and concentrated in vacuo. Purity was checked by HPLC.

### 2.4 Determination of total free thiol groups with Ellman's reagent

Jurkat cells were treated with 100 µM allicin for 10 min, harvested by centrifugation and washed in 100 mM sodium-phosphate buffer, pH 7.5. After centrifugation (600×*g*; Megafuge 1 OR, Heraeus, Hanau, Germany) cells were resuspended in 1 mL phosphate buffer, glass beads were added and centrifuged for 1 min to lyse the cells. Cell lysate (100 µL) was mixed with 900 µL of a 5 mM solution of Ellman's reagent [5,5′-Disulfanediylbis(2-nitrobenzoic acid)], DTNB; Sigma-Aldrich, Steinheim, Germany) and measured in a spectrophotometer (DU800, Beckman Coulter, Krefeld, Germany) at 412 nm.

### 2.5 Glutathione (GSH) determination

Cells were cultivated as described and counted in a Neubauer chamber and adjusted to the same cell number (2 × 10^7^). After washing in phosphate buffer (80 mM) containing 3.5 mM EDTA, cells were lysed by vortexing with glass beads and centrifuged at 15,800×*g* for 1 min. The supernatant was used to measure the GSH content according to Griffith [29]. The reaction mixture contained 12.5 µL supernatant, 5 µL GR (20 units mL^−1^; Sigma-Aldrich, Steinheim, Germany), 50 µL of 6 mM DTNB (Ellman's reagent, Sigma-Aldrich, Steinheim, Germany), and 350 µL of 0.3 mM NADPH (Carl Roth, Karlsruhe, Germany) in phosphate buffer. Distilled water was added to a final volume of 750 µL (final phosphate buffer 80 mM, 3.5 mM EDTA). The increase of optical density at 412 nm was measured in intervals of 30 s over 10 min using a spectrophotometer (DU800, Beckman Coulter GmbH, Krefeld, Germany).

### 2.6 Identification of *S*-thioallylated proteins using LTQ-Orbitrap mass spectrometry

Jurkat cells were treated with 100 µM allicin for 10 min, harvested by centrifugation and washed in 100 mM sodium-phosphate buffer, pH 7.5. After centrifugation (600×*g*; Megafuge 1 OR, Heraeus), cells were resuspended in 1 mL phosphate buffer containing 100 mM N-ethylmaleimide (NEM). For identification of *S*-thioallylated peptides, NEM-alkylated protein extracts were prepared from cell lysates of allicin-treated Jurkat cells. The protein extracts were separated by 15% non-reducing SDS-PAGE followed by tryptic in-gel digestion and LTQ-Orbitrap-Velos mass spectrometry as described [30]. Post-translational thiol-modifications of proteins were identified by searching all MS/MS spectra in “dta” format against the human proteome target-decoy protein sequence database (52,496 entries) extracted from UniprotKB release 12.7 using Sorcerer™-SEQUEST^®^ (Sequest v. 2.7 rev. 11, Thermo Electron including Scaffold 4.0, Proteome Software Inc., Portland, OR). The SEQUEST search parameters were used as described previously [30]. The Sequest search was carried out considering the following parameter: a parent ion mass tolerance 10 ppm, fragment ion mass tolerance of 1.00 Da. Up to two tryptic miscleavages were allowed. Methionine oxidation (Met+15.994915 Da), cysteine N-ethylmaleimide modification (Cys+125.04767 Da) and *S*-thioallylation (Cys+72.00337 Da for C_3_H_5_S_1_) were set as variable modifications. The Scores and mass deviations of the identified *S*-thioallylated peptides of the 332 proteins with the 72 Da mass shift on Cys residues are shown in Table S1 including their fragmentation spectra and fragment ion tables. The mass spectrometry proteomics data have been deposited to the ProteomeXchange Consortium via the PRIDE [31] partner repository with the dataset identifier PXD010201.

### 2.7 Flow Cytometric Measurement of Intracellular Free Zn^2+^ with FluoZin-3AM

1 × 10^6^ EL-4 T-cells were incubated with a final allicin concentration of 25 µM for 30 min with gentle shaking at 37 °C in the dark. Afterwards, cells were washed and loaded with 1 mL measurement buffer [32] for 30 min, containing 1 µM FluoZin-3AM (Thermo Fisher, Germany) and again gently shaken at 37 °C in the dark. Control cells were not treated with allicin. Cells were washed, resuspended in measurement buffer and incubated at 37 °C for 10 min either left untreated or further supplemented with either *N*,*N*,*N*’,*N*’-tetrakis(2-pyridylmethyl)ethylenediamine (TPEN, 50 µM) to obtain minimal fluorescence or with a combination of zinc sulfate (ZnSO_4_) and pyrithione (100 µM/50 µM) (all Sigma-Aldrich, Germany) to obtain maximal fluorescence. Subsequent flow cytometry measurements were performed using FACSCalibur (BD, Germany). Calculation of intracellular labile zinc was performed as described before [33] using the dissociation constant K_D_ = 8.9 nM for the FluoZin-3/Zn^2+^ complex [34].

### 2.8 IL-2 Quantification

For IL-2 determination, supernatants were harvested from 1 × 10^6^ cells/mL. In brief, EL-4 T-cells were either left untreated or were stimulated with allicin (25 µM) for 30 min at 37 °C. After 30 min, cells were washed and the medium was replaced with fresh EL-4 culture medium. In a next step, these cells were either left unstimulated or were stimulated for another 24 h at 37 °C with IL-1β (0.5 ng/mL, PeproTech, Germany). Subsequently, supernatants were taken and stored at −20 °C until ELISA measurement was performed using OptEIA mouse IL-2 ELISA (BD, Germany) according to the manufacturer's instructions.

### 2.9 Phalloidin-staining

Fibroblasts were grown in glass Petri dishes (∅ 5.5 cm) for three days in RPMI1640 medium as described above. Subsequently, the medium was removed and 2 mL PBS buffer was filled into the dishes. Allicin was added to a final concentration of 100 µM, 10 µM, 1 µM or 0.1 µM and cells were incubated at 37 °C and 5% CO_2_ for 10 min. As control, water was added in the same volume as allicin solution. Cells were twice washed with PBS buffer and fixed by adding 2 mL of 3.7% formaldehyde (Carl Roth, Karlsruhe, Germany) in PBS at room temperature. Cells were washed again twice again with PBS to remove the formaldehyde. To permeabilize the cells, 2 mL of 0.1% Triton-X 100 (Applichem, Darmstadt, Germany) in PBS were added to the cells for 5 min at room temperature, followed by repeated washing with PBS. To stain the cells with phalloidin-Rhodamine (Aatbioquest, Sunnyvale, CA, USA) in DMSO, the reagent was added according to the manufacturer's instruction and incubated for 20 min in the dark. Nuclei were counterstained using DAPI (1 µg/mL in methanol). After removal of the staining solution and washing, mounting medium was added (Roti®-Mount FlourCare, Carl Roth, Karlsruhe, Germany). Microscopy was performed using a Leica-fluorescence microscope (DM-RBE, Leica GmbH, Wetzlar, Germany), equipped with a rhodamine-filter (Em 590 nm, 20 nm bandwidth).

### 2.10 Enolase enzyme activity assay

The measurement of enolase activity was performed as described in Muller et al. [35]. Jurkat cells (about 2 × 10^8^) were harvested by centrifugation (300×*g*, 5 min, Heraeus megafuge 1OR), decanted and resuspended in PBS. Allicin was added to a final concentration of 100 µM, and washed by repeated centrifugation. Control cells were treated with distilled water instead of allicin solution. The pellet was resuspended in lysis buffer (20 mM TRIS, pH 7.5 and 1 mM EDTA [Carl Roth, Karlsruhe, Germany]). Cell lysis was performed by vortexing with glass beads for 1 min followed by centrifugation (5000×*g*, 2 min, 4 °C) and transfer of the lysate to a new tube. Protein concentration was measured using Bradford reagent [36].

Enolase activity was measured in 850 µL reaction buffer containing 100 mM 2,2',2′'-Nitrilotri(ethan-1-ol) (triethanolamine, Merck KGaA, Darmstadt, Germany), 0.2 mM NADH (Carl Roth, Karlsruhe, Germany), 30 mM MgSO_4_ (Carl Roth, Karlsruhe, Germany), 120 mM potassium chloride (Carl Roth, Karlsruhe, Germany) and 1.75 mM adenosine diphosphate [ADP] (Merck, Darmstadt, Germany). The components were mixed in a 1 mL cuvette with 50 µL substrate solution (45 mM 2-phosphoglycerate, Sigma Aldrich, Steinheim, Germany), 50 µL cell lysate and 50 µL enzyme mix (200 U/mL pyruvate kinase and 300 U/mL lactate dehydrogenase) (Merck, Darmstadt, Germany). One unit was defined as 1 µmol substrate turnover per minute. Extract buffer (50 µL) was used as blank. Decrease of light absorbance at 340 nm was measured over 7 min in a Beckman spectrophotometer (DU 800, Beckman Coulter, Krefeld, Germany). To calculate the enzyme activity, a molar extinction coefficient for NADH at 340 nm of 3400 Lmol^−1^cm^−1^ was used [37].

## 3 Results and discussion

### 3.1 Allicin causes global *S*-thioallylation in the proteome of Jurkat T- cells

**Fig. 2:**
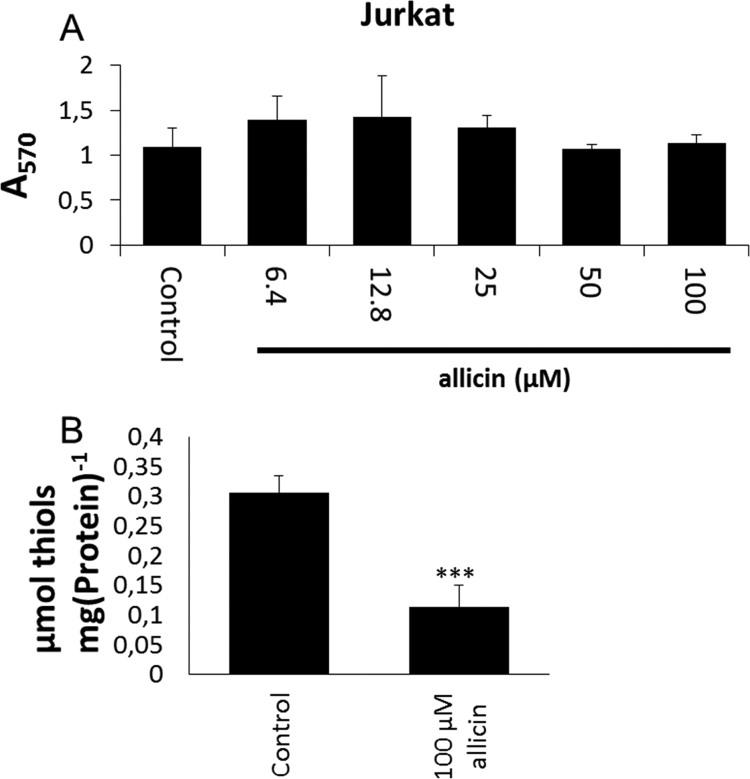
Calibration for Jurkat T-lymphocyte cells with allicin. (A) The metabolic activity of Jurkat T-cells was not affected by cultivation with ≤ 100 µM allicin. Metabolic activity was determined by MTT staining after 24 h. Bars indicate standard deviation with n = 3 independent experiments each with 3 technical replicates. (B) Jurkat T-cells were treated with 100 µM allicin for 10 min resulting in approximately 50% reduction in the total free thiols as determined by titration with Ellman's reagent. Error bars indicate standard error of the mean, the *** symbol indicates *P* < 0.001 (Student's *t*-test), data are for three independent experiments each with three technical replicates.

First, we determined the metabolic activity of Jurkat cells in the presence of various concentrations of allicin using a standard MTT test. The results showed that the metabolic activity of Jurkat cells remained comparable to controls after 24 h exposure of up to 100 µM allicin (Fig. 2A). Next, we monitored the effect of allicin on free sulfhydryl groups in crude cell lysates using Ellman's reagent which was normalized to the protein content.

The goal was to establish conditions for the proteomics study to identify primary allicin targets in Jurkat cells. Treatment of Jurkat cells with 100 µM allicin for 10 min led to a 50% decrease in free cellular thiol groups from approximately 0.27 µmol mg^−1^ protein to 0.13 µmol mg^−1^ protein (Fig. 2B). This confirmed that allicin reacts efficiently and rapidly to modify cellular thiols and the short treatment time was chosen to identify primary effects on cellular proteins rather than slower, adaptive responses. Adaptation of cells to sublethal doses of allicin manifests for example in the recovery of growth of microorganisms in culture, with a dose-dependent length of the lag phase [38], [39], [25].

**Fig. 3:**
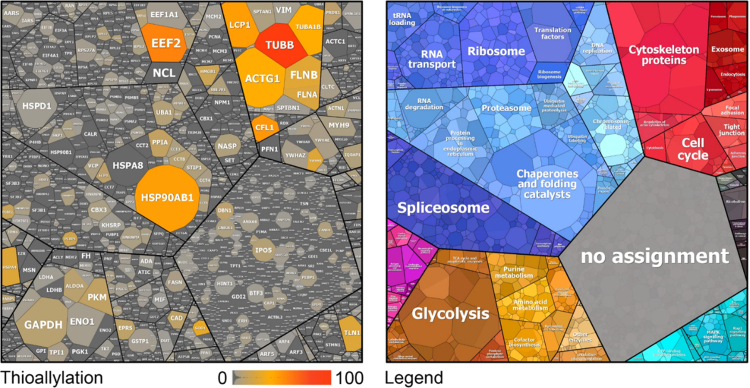
In total, 332 proteins were modified by *S*-thioallylation in the Jurkat cell proteome which are displayed in a proteome abundance treemap. The cell size of the *S*-thioallylation treemap (left) indicates the protein abundance of 2177 proteins in the proteome of Jurkat cells as revealed by spectral counting using the Scaffold proteome software. The colour code denotes the abundance of *S*-thioallylated peptides for the identified proteins as detected using spectral counts. Quantification was performed using the Scaffold proteome software from 3 biological replicates. The proteins were classified according to KEGG ontology annotation into different functional categories listed in Table S3 and shown in the treemap legend (right). Of note, the most abundant *S*-thioallylated proteins are also the most abundant thiol-containing proteins including chaperones (HSP90, HSP4A), the cytoskeletal proteins actin, tubulin, filamin, cofilin, plastin-2 (ACTG, TUBB/A, CFL1, FLNA/B, LCP1) and elongation factors (EEF2).

**Fig. 4:**
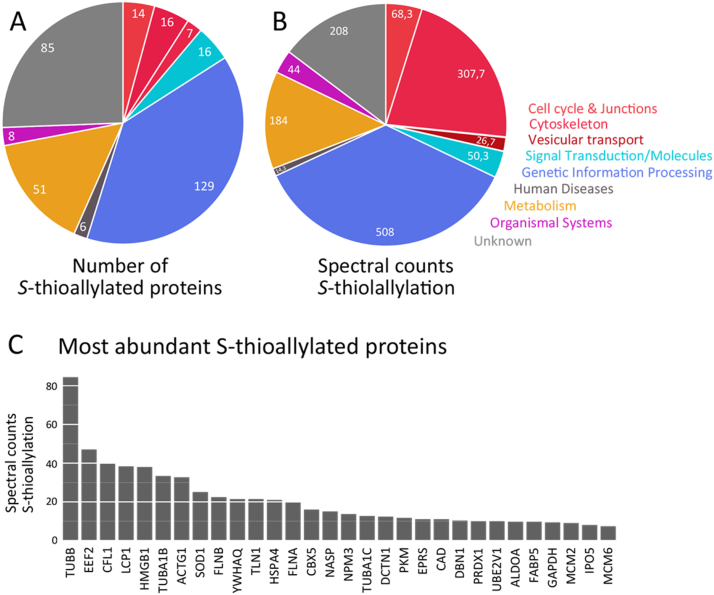
Total number (A) and spectral counts (B) of 332 proteins with S-thioallylation identified in the human Jurkat cell proteome. (A) The proteins with *S*-thioallylations were counted based on their functional classification by KEGG ontology. (B) The spectral counts of all *S*-thioallylated proteins are counted for each KEGG category. (C) The most abundantly *S*-thioallylated proteins are shown based on their spectral counts of *S*-thioallylated peptides. The *S*-thioallylated proteins are classified in Table S1 into their functional categories.

Allicin has been previously shown to cause *S*-thioallylation of several Cys peptides in the proteome of *E. coli* cells [25]. Here we were interested to identify the extent of protein S-thioallylation in the proteome of Jurkat cells after exposure to 100 µM allicin for 10 min. Tryptic peptides of the whole Jurkat cell proteome were subjected to shotgun Orbitrap LC-MS/MS analysis. The Cys peptides identified in the Jurkat cell proteome were searched for a mass shift of 72 Da after allicin treatment. In total, 2177 proteins were identified in three biological replicates in the Jurkat cell proteome using the Scaffold software and quantitative values were calculated based on their spectral counts (Table S2). These 2177 total proteins included 332 proteins with *S*-thioallylated peptides of different spectral counts (Table S1). The *S*-thioallylated proteins could be allocated to all KEGG (Kyoto Encyclopaedia of Genes and Genomes) ontology categories, including cellular metabolism, genetic information processing, translation, cytoskeleton, cell cycle, splicing and protein quality control. (Table S1, Fig. 3). The 332 proteins that are modified by *S*-thioallylation in the total Jurkat cell proteome are shown in the protein abundance treemap [40] in Fig. 3. All 2177 identified proteins were sorted according to the KEGG functional categories in this proteome abundance treemap based on their total spectral counts and those with *S*-thioallylations were marked with a colour code according to the number of *S*-thioallylated peptides. Proteins with most abundant protein *S*-thioallylations were found in the KEGG categories cytoskeleton (16 proteins with an average of 308 peptides), genetic information processing (129 proteins with 508 peptides), cellular metabolism (51 proteins with 184 peptides) and unknown functions (85 proteins with 208 peptides) (Fig. 4A, B). Further proteins with *S*-thioallylation could be allocated to the categories cell cycle and junctions (14 proteins with 68 peptides), vesicular transport (7 proteins with 27 peptides), signal transduction (16 proteins with 50 peptides), human diseases (6 proteins with 14 peptides) and organismal systems (8 proteins with 44 peptides). Of note, the most abundant *S*-thioallylated proteins are often also the most abundant proteins in the proteome of Jurkat cells (Fig. 3).

The distribution of the 332 *S*-thioallylated proteins in different functional categories is shown in Fig. 4A and B. The most abundant *S*-thioallylated proteins include cytoskeleton proteins, such as actin and tubulin (ACTG, TUBB, TUBA1), plastin-2 (LCP1), filamin (FLNA/B) and cofilin-1 (CLF1) and are detailed in Fig. 4C. In addition, abundant *S*-thioallylated proteins were identified as heat shock chaperones (HSP90, HSPA4), elongation factors (EEF2), high mobility group protein B1 (HMGB1) and other DNA maintenance proteins, the glycolytic fructose-bisphosphate aldolase A (ALDOA), glyceraldehyde 3-phosphate dehydrogenase (GAPDH) and pyruvate kinase (PKM) as well as the [Cu-Zn]-superoxide dismutase (SOD1) and peroxiredoxin-1 (PRDX1) involved in antioxidant defence (Fig. 4C, Table S1).

### 3.2 Allicin disrupts the actin cytoskeleton and alters cell morphology

**Fig. 5:**
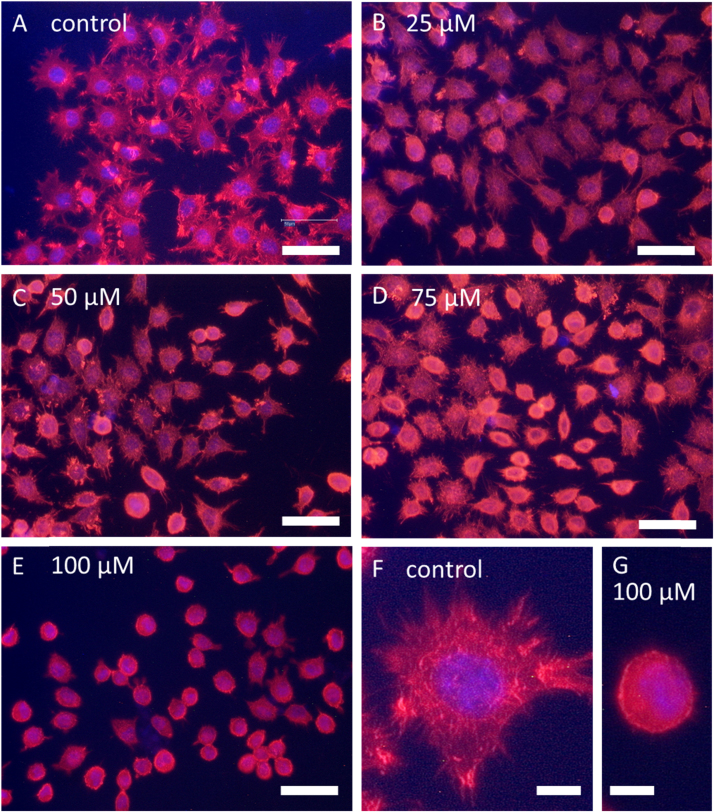
Effect of allicin on the actin cytoskeleton and cell morphology of mouse fibroblasts. Mouse L929 fibroblasts (A, F) were exposed to different concentrations of allicin (B-E, G) for 10 min and stained with rhodamine-phalloidin to visualize the change in the actin cytoskeleton. DAPI was used to stain the nuclei. The stained fibroblasts are shown before (A) and after treatment with 25 µM (B), 50 µM (C), 75 µM (D) or 100 µM allicin (E) for 10 min. Single cells are displayed at a higher magnification under control conditions (F) and after treatment with 100 µM allicin (G). The cytoplasmic branches of the actin cytoskeleton are completely lost in allicin-treated cells, which develop spherical cellular morphologies. The actin filaments in the fibroblasts become amorphous, which indicates a strong effect of allicin on the actin cytoskeleton. A-E scale bar = 50 µm, F-G scale bar = 10 µm.

Next, we were interested if the widespread observed *S*-thioallylation in the proteome affects cellular functions. Thus, we investigated the functions of selected allicin-modified proteins after allicin treatment. In the category cytoskeleton, 16 proteins were identified as most abundantly *S*-thioallylated with 308 total spectral counts (Table S1). These *S*-thioallylated cytoskeleton proteins included actin (ACTG), tubulin (TUBB, TUBA1), filamin (FLNA, FLNB), the actin binding proteins cofilin (CFL1), plastin-2 (LCP1), the Arp2/3 actin remodelling complex (ARPC5), and the actin capping complex (CAPZB) (Table S1, Fig. 4). The actin cytoskeleton is important for cell morphology and can be easily visualized with phalloidin-based fluorescent dyes which stain filamentous actin (F-actin). We used L929 murine fibroblasts to study the effect of allicin on the cytoskeleton and cell morphology. Untreated fibroblasts showed a branched cytoplasm (Fig. 5A) with clearly visible rhodamine-phalloidin stained actin filament cables (Fig. 5F). Upon treatment with 25–100 µM allicin for 10 min, we observed a dose-dependent rounding-up of the cells with concomitant loss of actin filaments (Fig. 5B-E, G). Furthermore, the nucleus was less well defined by staining allicin-treated cells with DAPI (Fig. 5A-G). These effects are presumably due to the oxidative *S*-thioallylation of actin and the actin-binding proteins which regulate the assembly and disassembly of actin filaments [41], [42], [43]. Thus, cofilin speeds up actin polymerization via its actin-severing activity which provides free barbed ends for further polymerization and nucleation by the Arp2/3 complex. The LCP1 protein also contains an actin-binding domain [44] and acts as a calcium-dependent actin-bundling protein [45]. LCP1 is also important for crosslinking and stabilization of F-actin structures [46]. Although cytoskeleton proteins were among the most abundantly *S*-thioallylated proteins, the mechanism of actin cytoskeleton disruption remains to be elucidated. However, there seem to be parallels to the reversible depolymerization: polymerization of actin filaments by glutathionylation: deglutathionylation, respectively [47], [48].

In a previous study it was shown that allicin caused microtubule depolymerization and inhibition of tubulin polymerization in mouse NTH-3T3 fibroblasts [49]. However, no effect on the actin cytoskeleton was observed after treatment with low doses of 0.5 µM allicin for 30 min. The authors concluded that allicin inhibited cell division by disrupting spindle formation during mitosis [49]. However, exposure to higher concentrations of 25 µM allicin for one hour resulted in inhibition of actin polymerization in human T-cells. In addition, SDF-1α-induced T-cell adhesion to fibronectin and endothelial cells was inhibited by higher allicin doses [22]. The cytoskeleton is a major target for innovative cancer therapies [50], [51], [52] and the high number of allicin targets in this category of cellular proteins merits further investigation in this regard.

### 3.3 Allicin leads to Zn^2+^ release in murine EL-4 cells

**Fig. 6:**
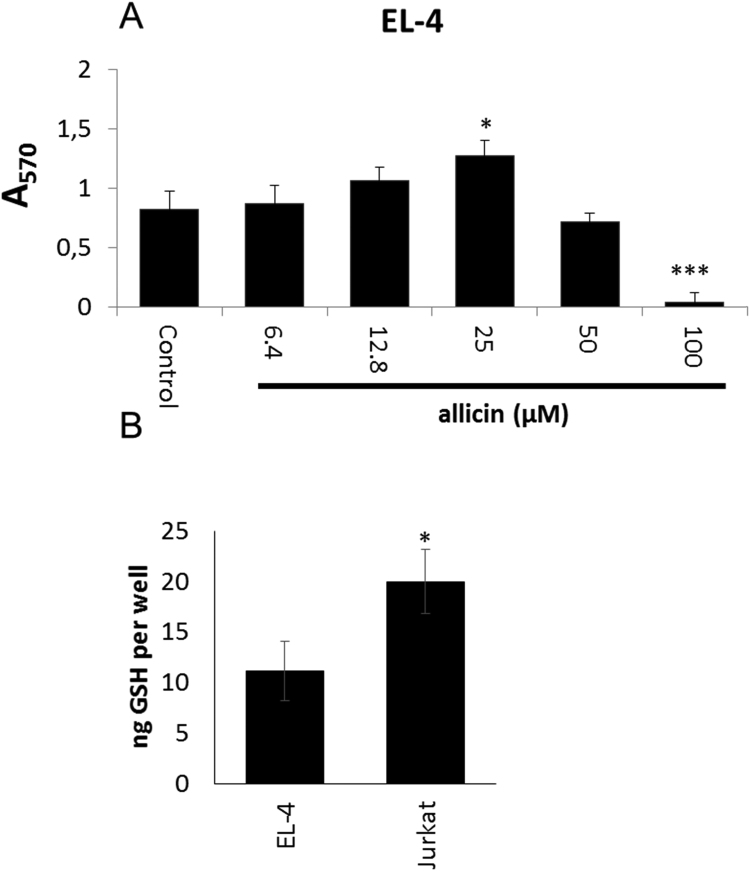
The high sensitivity of EL-4 cells to allicin is probably related to their low GSH content. (A) Murine EL-4 T-cells were cultivated in the presence of various allicin concentrations and the metabolic activity was determined by MTT staining after 24 h. Bars indicate standard error of the mean, n = 3 independent experiments each with 3 technical replicates. (B) GSH was measured in a glutathione reductase cycling assay [28]. Data for 5 independent experiments each with 3 technical replicates. * *P* < 0.05, ***P < 0.001 (Student's *t*-test).

Zn^2+^ is the second most abundant trace element in humans and an essential cofactor for many enzymes. Zn^2+^ is required for the correct function of the immune system and plays an important role in immune signalling [53]. Cysteine and histidine residues in proteins function as Zn^2+^ binding ligands in cellular proteins [54]. Oxidative stress can lead to oxidation of protein thiols followed by Zn^2+^ release. Allicin-induced Zn^2+^ release has already been demonstrated in macrophages [23]. A major quantity of intracellular Zn^2+^ is bound by [Cu,Zn] superoxide dismutase in human cells [55]. In this work, SOD1 was identified as major target for *S*-thioallylation in response to allicin (25 spectral counts) (Table S1, Fig. 4). Therefore, we investigated the effect of biocompatible allicin treatment on immune signalling in murine EL-4 T-cells. An MTT assay of metabolic activity showed that EL-4 cells were more sensitive to allicin than Jurkat cells (Fig. 6A). Thus, we treated EL-4 cells with lower doses of biocompatible 25 µM allicin which did not affect metabolic activity. In view of what is known about the importance of GSH and GSH metabolism for the resistance of cells to allicin, the differential sensitivity of EL-4 and Jurkat cells is probably related to the much lower levels of GSH found in EL-4 cells (Fig. 6B).

**Fig. 7:**
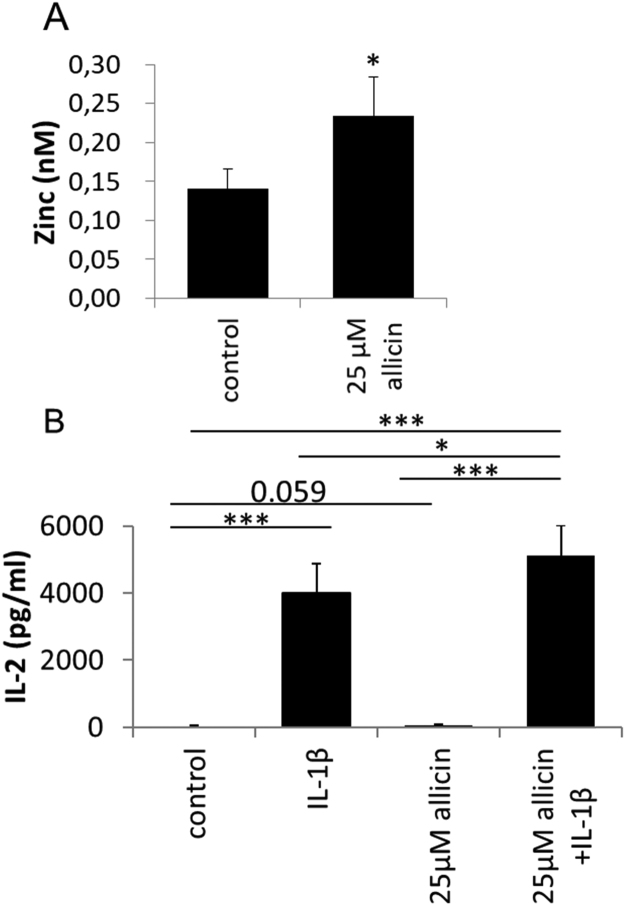
Allicin leads to Zn^2+^ release in murine EL-4 cells and stimulates IL-1β dependent synthesis of IL-2. (A) By 30 min after exposure to 25 µM allicin an enhanced level of intracellular Zn^2+^ was measured in EL-4 cells. Bars indicate standard error of the mean, n = 5 independent experiments. *P < 0.05. (B) IL-1b stimulated synthesis of IL-2 measured 24 h after treatment was significantly higher in allicin-treated cells. Bars indicate standard error of the mean, n = 11–12 independent experiments. * *P* < 0.05, ***P < 0.001.

Interleukin-1 (IL-1) activates transcription of the interleukin-2 gene, leading ultimately to the release of IL-2 protein by EL-4 T-cells. This process is known to be Zn^2+^-dependent [56]. An enhanced level of free Zn^2+^ (*P* < 0.05) was measured in murine EL-4 T-cells 30 min after exposure to 25 µM allicin (Fig. 7A). In turn, 24 h after IL-1 treatment the higher levels of intracellular Zn^2+^ resulted in an increased production of IL-2 in the allicin-treated EL-4 cells. This demonstrates clearly that allicin at biocompatible doses over an extended period can lead to important physiological changes, such as stimulation of the immune system (Fig. 7B). Immunomodulatory effects of garlic compounds have been described *in vitro* and *in vivo* [57], [58]. However, molecular mechanisms were so far missing. Reduced IL-2 production by T-cells is frequent in elderly patients [59] suffering from systemic lupus erythematosus [60] and other dysregulations and impairments of the immune system.

### 3.4 Allicin *S*-thioallylates several glycolytic enzymes and inhibits enolase activity in Jurkat cells

**Fig. 8:**
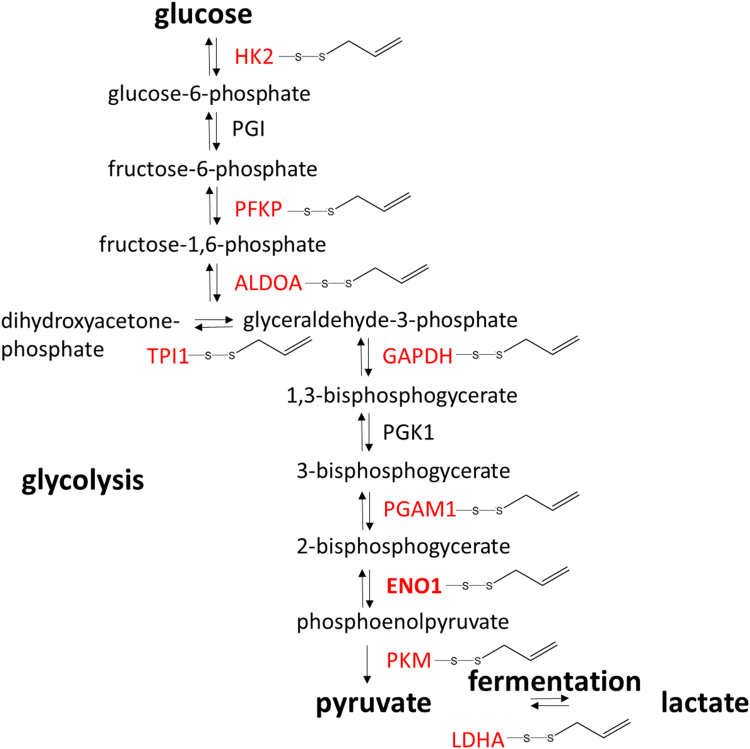
Enzymes of glycolysis and fermentation *S*-thioallylated in Jurkat cells by allicin (shown in red). ALDOA = fructose bisphosphate aldolase, ENO1 = enolase, GAPDH = glyceraldehyde-3-phosphate dehydrogenase, HK2 = hexokinase, LDHA = lactate dehydrogenase A, PFKP = phosphofructokinase, PGAM1 = phosphoglycerate mutase, PGI = phosphoglucose isomerase, PGK1 = phosphoglycerate kinase, PKM1 = pyruvate kinase. (For interpretation of the references to color in this figure legend, the reader is referred to the web version of this article).

Although normal cells mostly produce their cellular energy via oxidative phosphorylation in the mitochondria, in the well-known Warburg effect cancer cells predominantly produce their energy through increased glycolysis followed by lactic acid fermentation. The catabolism of glucose to pyruvate is catalysed by 10 enzymes, 8 of which are *S*-thioallylated by allicin in Jurkat cells. Furthermore, lactate dehydrogenase which converts pyruvate to lactate is also an allicin target (Fig. 8, Table S1).

**Fig. 9:**
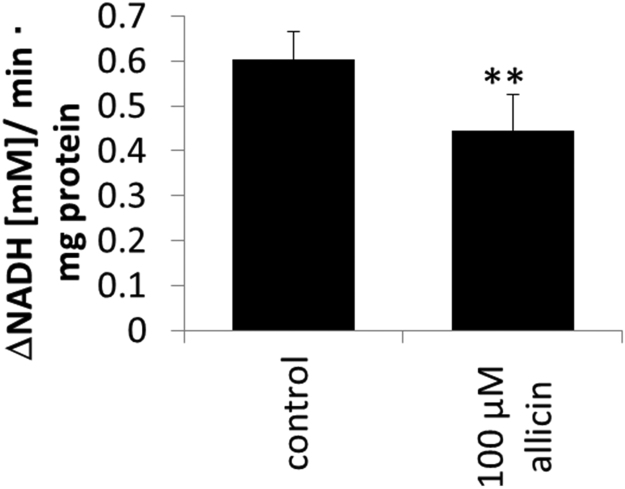
Inhibition of enolase-activity in Jurkat cells after treatment with 100 µM allicin for 10 min. Enolase activity was monitored in cell lysates using a spectrophotometric assay monitoring NADH consumption as absorbance change at A_340_ nm. The assay mixture included cell lysate, the substrate 2-phosphoglycerate, the enzymes PEP kinase and lactate dehydrogenase and NADH. Error bars show standard deviation. The ** symbol denotes a statistically significant difference between control and allicin treatment (Student's *t*-test, *P* < 0.01) with n = 3 technical replicates. The experiment was repeated twice with similar results.

Enolase has a sulfhydryl group in its catalytic centre [61] and is an enzyme of the glycolytic pathway converting 2-phosphoglycerate to phosphoenolpyruvate (PEP). The human genome encodes three isoforms of enolase. The non-neuronal enolase (NNE, alpha enolase, ENO1) is expressed in many different cell types, including Jurkat cells. The beta-enolase gene is mainly expressed in muscles and the gamma enolase predominantly in neurons [62]. ENO1 is the most abundant enolase found in nearly all tissues. We chose ENO1 for functional investigation because it was identified as a minor allicin target with only 3 spectral counts (Table S1). We were interested if allicin affects also the enzyme activity of quantitatively less *S*-thioallylated proteins. Jurkat cells were exposed to 100 µM allicin for 10 min and enolase activity was monitored in cell lysates using a spectrophotometric assay at A_340_ nm by following 2-phosphoglycerate-dependent changes in NADH consumption. In brief, enolase in the cell lysate converts 2-phosphoglycerate to phosphoenolpyruvate (PEP) and the added PKM and LDHA included in the assay convert PEP to pyruvate and subsequently to lactate resulting in NADH consumption which is measured spectrophotometrically at A_340_. Allicin treatment caused a reduction of enolase activity (*P* < 0.01) from ~0.6 mM NADH min^−1^ mg^−1^ protein in untreated control cells to ~0.45 mM NADH min^−1^ mg^−1^ protein in allicin-treated cells (Fig. 9). The prominent *S*-thioallylation of glycolytic enzymes, such as ENO1, ALDOA and PKM (Fig. 8, Table S1) suggests that allicin can inhibit glycolysis which provides electron donors for ATP generation required for cellular biosynthesis pathways and growth of the cells. Enolase also acts as a plasminogen receptor and mediates the activation of plasmin and extracellular matrix degradation [63]. In tumour cells, *ENO1* is up-regulated and supports the Warburg effect. ENO1 is expressed at the cell surface where it promotes cancer invasion, and is subjected to a specific array of post-translational modifications, namely acetylation, methylation and phosphorylation. Enolase is a tumour-associated antigen (TAA) and ENO1 overexpression and post-translational modifications could be of diagnostic and prognostic value in many cancer types. Enolase is a target for tumour therapy and DNA vaccination with ENO1 in preclinical models efficiently delayed the development of very aggressive tumours such as pancreatic cancer [63]. The observation that allicin leads to *S*-thioallylation of ENO1 opens up potential avenues for developing cancer therapies. The investigation of allicin's effects on other glycolytic enzymes in view of the Warburg effect, is a high priority [64].

### 3.5 Bioavailability of allicin

The results documented in this report are for cells directly exposed to allicin. While the effects of allicin on protein *S*-thioallylation are impressive, the question arises about the bioavailability of allicin after garlic consumption, a topic which has been addressed in several studies [65]. It is generally considered that garlic consumption promotes health and it has been implicated in positive effects on a number of conditions such as various cancers, diabetes, and heart disease [1]. However, allicin ingested orally is rapidly hydrolysed in the stomach to 2-propenethiol which is quickly metabolised to allyl methyl sulfide, both of which are major components of garlic breath and are also excreted in the urine [66], [67], [68]. These reactions compete with the reaction of allicin with thiols, and reaction of allicin with glutathione will further reduce the amount remaining for *S*-thioallylation of cysteine residues in proteins. For these reasons, achieving therapeutically relevant concentrations of allicin via the oral route is therefore unlikely and more direct routes of application to the desired site of action need to be considered. For example, a strategy to generate allicin *in situ* within tumour tissue was used effectively against a human tumour cell line xenograft in athymic nude mice, while at the same time leaving other tissues unharmed [20]. Topical application of allicin for skin tumours or direct inhalation via the pulmonary route need to be investigated. Despite these pragmatic considerations regarding the development of successful treatments with allicin, our data provide novel insights into the redox-active mode of action of allicin inside human cells. Additionally, although our study was performed with pure allicin, this represents generally about 60–80% of the total thiosulfinates formed in garlic tissues upon wounding [69]. Other sulfinates might be expected to react with protein cysteines similarly to allicin and would lead to characteristic mass shifts in the affected peptides.

### 3.6 Reversibility of allicin-mediated *S*-thioallylation

The *S*-thioallylation reaction of a protein thiol with allicin can be likened to a thiol-disulfide exchange reaction. However, the polarized bond between the *O*-atom and one of the *S*-atoms in allicin enhances the electrophilic nature of the *S*-atom and makes it more reactive than a simple disulfide towards the nucleophilic thiol group, and in contrast to straightforward thiol-disulfide exchange, the electrons end up in water. Nevertheless, the resulting disulfide bond can presumably be reduced back to a thiol similarly to any other protein disulfide, for example by thioredoxins or glutaredoxins, or potentially even by glutathione reductase. In support of this notion, we have recently shown that *S*-allylmercaptoglutathione, produced when allicin reacts with the thiol group in glutathione, is a substrate for glutathione reductase [70].

### 3.7 Conclusions and perspective

• Biocompatible allicin doses led to *S*-thioallylation of 332 proteins in the human Jurkat cell proteome, showing its strong effect on mammalian cells.
• Selected *S*-thioallylated proteins were confirmed to be inhibited by allicin as part of the molecular mechanism of garlic action leading to disruption of the actin cytoskeleton, a decrease in enolase activity and enhanced Zn^2+^ release modulating the immune system.
• There are several reports of the benefit of garlic consumption in relation to cancer protection and the high proportion of allicin targets in the cytoskeleton and in glycolysis are potentially indicative of mechanisms by which this might function.
• In sum, these data help to elucidate the mode of action of allicin, commonly consumed in the popular foodstuff garlic.
